# Impact of Overweight on Spatial–Temporal Gait Parameters During Obstacle Crossing in Young Adults: A Cross-Sectional Study

**DOI:** 10.3390/s24237867

**Published:** 2024-12-09

**Authors:** Matthias Chardon, Fabio Augusto Barbieri, Clint Hansen, Pascal Petit, Nicolas Vuillerme

**Affiliations:** 1 AGEIS, Université Grenoble Alpes, 38000 Grenoble, France; matthias.chardon@univ-grenoble-alpes.fr (M.C.); c.hansen@neurologie.uni-kiel.de (C.H.); pascal.petit@univ-grenoble-alpes.fr (P.P.); 2 Human Movement Research Laboratory (MOVI-LAB), Department of Physical Education, São Paulo State University (UNESP), Bauru 17011180, SP, Brazil; 3 Department of Neurology, Kiel University, 24105 Kiel, Germany; 4 Institut Universitaire de France, 75005 Paris, France

**Keywords:** overweight, obstacle crossing, gait, clearance

## Abstract

**Background**: Overweight may present an additional challenge when crossing obstacles. More specifically it may affect adequate foot clearance to reduce the risk of obstacle contact. Thus, the objective of this study was to compare obstacle clearance and spatial–temporal gait parameters during obstacle crossing in young adults with normal body weight and overweight. **Methods**: Twenty-eight and fifteen individuals were categorized into normal body mass index (18.5–25 kg/m^2^) and overweight (25–30 kg/m^2^), respectively. The participants walked along a walkway at their preferred speed and stepped over an obstacle. Spatial–temporal parameters were calculated during the approaching (stride before obstacle) and the crossing (step over the obstacle) phases. Additionally, the leading and trailing foot placements prior to and after the obstacle and toe clearance were calculated. **Results**: No significant differences were found for the approach, the crossing phases and leading and trailing toe clearance. Analysis of foot placement distance prior to and after the obstacle showed that, compared to the individuals with normal body weight, overweight individuals significantly increased the leading foot placement distance prior to the obstacle (+7 cm, ↑ 6.7%) and increased the trailing foot placement distance after the obstacle (+8.1 cm, ↑ 9%). **Conclusions**: Our findings indicated that overweight individuals have a different obstacle crossing behavior regarding foot placement distance prior to and after the obstacle compared to normal-weight individuals without differences in spatial–temporal gait parameters or toe clearances. However, the results did not suggest that participants with overweight show a higher risk of tripping.

## 1. Introduction

Overweight and obesity have become a significant problem worldwide, with the obesity prevalence having nearly tripled in the last 50 years [1]. The body mass index (BMI) is commonly used to assess overweight (BMI between 25 and 29.9 kg/m^2^) and obesity (BMI ≥ 30 kg/m^2^). Overweight and obesity impose functional limitations by the additional loading of the increased body mass to the locomotor system, resulting in gait alterations and aberrant mechanism in posture and balance [2,3,4,5,6,7,8]. Although the question of whether and how overweight and obesity could affect gait during unobstructed walking in adults has received a large interest in the past few decades (for recent reviews, see [9,10,11]), not much is reported about how overweight and obesity could impact gait under more challenging conditions, such as crossing an obstacle while walking. However, it is probable that excess body weight may present an additional challenge when crossing obstacles and ensuring adequate foot clearance. A systematic review on the influence of overweight and obesity on obstacle crossing performance [12] identified a small number of published studies that have specifically compared obstacle crossing during walking in obese or overweight adults to those with normal body weight (*n* = 3) [13,14,15]. Despite this small number of published studies and their heterogeneity, the results of this review suggested that overweight and obesity significantly impacted gait patterns during obstacle crossing in adults.

Compared to normal-body-weight adults, overweight or obese adults exhibit lower velocity [13,15], shorter step length, lower cadence, larger step width, less time spent in single-limb support, more time spent in double support [13] and a reduced swing time (as a percentage of the gait cycle) [15] during obstacle crossing. At this point, however, the three studies that investigated the effects of overweight or obesity on gait behavior during obstacle crossing have certain limitations; we were particularly concerned about two of them.

First, none of the studies examined the distance from the foot to the obstacle, which is called “horizontal and vertical foot–obstacle clearance distances” or “toe/heel clearance”. These parameters are usually considered as a critical parameter in a variety of obstacle crossing tasks (e.g., for reviews, see [16,17]). More largely, it is further recognized that since the foot clearance is the safety margin in the obstacle crossing task, decreasing the clearance might lead to a higher risk of tripping over an obstacle (e.g., [18,19,20]). In addition, foot placement prior to and after the obstacles may be fundamental, since errors may result in a misstep [21]. An optimal foot placement to the obstacle allows enough time to flex the limb and clear the obstacle [21], especially when body limitations are present as in obesity.

Second, none of the above-mentioned studies specifically compared obstacle crossing during walking between normal-body-weight (BMI of 18.5–24.9 kg/m^2^) vs. overweight individuals (BMI of 25.0–29.9 kg/m^2^). Therefore, potential differences in obstacle clearance and spatial–temporal gait parameters during obstacle crossing among overweight individuals remain unknown, even though differences compared to normal-weight individuals have been demonstrated during flat-ground walking [22].

Silva et al. (2017) [14] and Desrochers et al. (2021) [13] compared a group of normal-body-weight participants to a group of obese participants (BMI ≥ 27 [14] or 30 kg/m^2^ [13]), which according to the WHO classification combines both overweight and obese individuals (mean BMI: 29.9 ± 2.5 kg/m^2^) [23]. Gill (2019) [15] included the so-called “Overweight/Class I obesity” group (BMI of 25–34.9 kg/m^2^) that hence brought together overweight individuals (BMI of 25.0–29.9 kg/m^2^) and obese individuals (BMI of 30.0–34.9 kg/m^2^). All of the studies highlighted group differences in the spatial–temporal parameters when comparing the overweight and normal-body-weight participants.

Consequently, the aim of the present study is to compare obstacle clearance and spatial–temporal gait parameters during an obstacle crossing task in normal-body-weight and overweight young adults. We hypothesized that overweight impacts gait behavior during obstacle crossing, particularly on obstacle clearance parameters by reducing foot clearances, which might lead to an increased risk of tripping.

## 2. Materials and Methods

This study is a secondary analysis of previous studies performing obstacle crossing tasks [24,25,26]. Data from forty-three participants in these studies were analyzed. Only healthy participants aged 20–39 years were included in this analysis [24], specifically those in the healthy control group, excluding participants with multiple sclerosis [25,26]. No new data were collected for this specific analysis.

### 2.1. Participants

Based on the classifications for BMI recommended by the World Health Organization (WHO) [27], the 43 participants were categorized into two groups of (1) 28 (65%) individuals with normal body weight (BMI of 18.5 to <24.9 kg/m^2^) and (2) 15 (35%) overweight individuals (BMI of 25 to <29.9 kg/m^2^). Apart from their weight status, participants were considered healthy (i.e., no participant with multiple sclerosis was included). The subjects were informed about the experimental procedures (University Institution Review Board CAAE#99191318.0.0000.5398) and signed informed consent forms. Also, the experiment was performed in accordance with the Declaration of Helsinki and Brazilian ethical regulations.

### 2.2. Obstacle Crossing Task

Participants were instructed to walk barefoot at a self-selected speed along a 8.5 m long * 1.5 m [25] or 3.5 m [26] wide wooden pathway and to step over an obstacle (height = 15 cm, width = 80 cm, thickness = 2 cm, foam material [25]) without making contact, positioned in the middle of the pathway. The pathway was covered with a 3 mm thick black rubber carpet [24]. Three trials were performed. The starting point of each trial was adjusted to ensure that the obstacle was crossed with the right leg as the leading limb and that two strides were completed prior to the obstacle crossing.

### 2.3. Data Collection

Kinematic data were captured using a system of ten infrared cameras (Vicon Motion System^®^, Oxford, UK, 200 Hz). Two markers were attached at the top of the obstacle. Additional markers were positioned as follows: on the lateral aspect of the calcaneus and the head of the second metatarsus of the right limb, and on the medial aspect of the calcaneus and the head of the second metatarsus of the left limb. The limbs were conventionally labeled: the leading foot was defined as the first to cross the obstacle, and the trailing foot as the second. Gait signals were processed using a fifth-order low-pass Butterworth filter (6 Hz, zero-lag).

**Figure 1:**
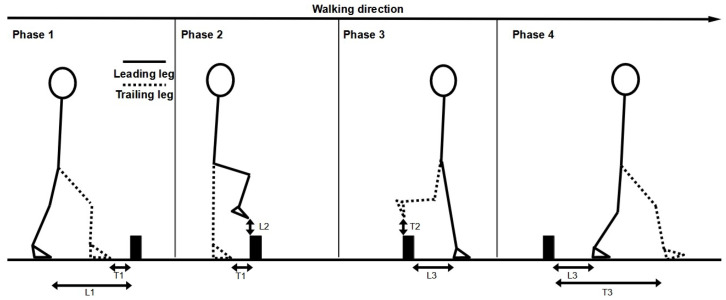
Description of the different phases of the obstacle crossing task and the obstacle clearance parameters. L1: leading foot horizontal distance prior to the obstacle (cm); L2: leading foot toe clearance (vertical distance between the foot marker and the obstacle) (cm); L3: leading foot horizontal distance after the obstacle (cm); T1: trailing foot horizontal distance prior to the obstacle (cm); T2: trailing foot toe clearance (cm); T3: trailing foot horizontal distance after the obstacle (cm).

Figure 1 illustrates the obstacle clearance outcomes evaluated. The following parameters were analyzed:

- Spatial–temporal gait parameters: stride length, width, duration, velocity and double-support time during the approach phase (stride before the obstacle) and the crossing phase (step over the obstacle);
- Leading and trailing foot placement prior to the obstacle (horizontal distance of the leading and trailing foot from the second metatarsal marker to the obstacle prior to crossing);
- Leading and trailing vertical toe foot clearance during obstacle crossing (vertical distance of the second metatarsal marker from the top of the obstacle for the leading and trailing foot during crossing);
- Leading and trailing foot placement after the obstacle (horizontal distance of the leading and trailing foot from the heel marker to the obstacle after crossing).

### 2.4. Statistical Analysis

All statistical analyses were performed using R software 4.2.2^®^ (R Core Team, Vienna, Austria) for Windows 10©. Vargha and Delaney’s A (VDA) was calculated using the effect size package [28]. For each participant, the mean trial value of each gait parameter was used. In a previous analysis, we demonstrated that gait and clearance parameters were reliable across three trials [29]. In addition, for data analysis, previous studies commonly used the mean of the three trials of gait outcomes in obstacle crossing tasks [30,31,32,33,34].

Due to skewed, non-normal or multimodal distributions, the outcomes were evaluated with nonparametric methods [35]. The Wilcoxon–Mann–Whitney rank sum test was used to test whether there were differences in socio-demographics, anthropometrics and gait parameters between the two groups. The Wilcoxon effect size (ES) and its 95% confidence interval were computed to quantify the difference between the two groups beyond *p*-value interpretation. To that end, the Vargha and Delaney’s A statistic (VDA) was used. VDA is a standardized quantification of the difference between the two groups. VDA indicates the likelihood that a randomly chosen value from one group exceeds a randomly chosen value from the other group. A value of 0.50 reflects stochastic equality between the two groups. A value of 1 signifies that the first group (individuals with normal body weight) consistently outperforms the second group (overweight individuals) in stochastic terms. A value of 0 indicates complete stochastic domination by the second group (overweight individuals). Vargha and Delaney (2000) suggested an ES of 0.45–0.55 as a negligible effect, 0.56–0.63 (or 0.35–0.44) as a small effect, 0.64–0.70 (or 0.30–0.34) as a medium effect and >0.70 (or <0.30) as a large effect [36]. The type I error rate was set at α = 0.05.

## 3. Results

### 3.1. Socio-Demographic and Anthropometric Characteristics

Table 1 presents the results from the Wilcoxon–Mann–Whitney rank sum tests examining differences between the two groups.

Age and the body height did not significantly differ between the two groups (*p* > 0.05), while body mass and BMI did. Overweight individuals had a significantly higher body mass (*p* < 0.01) and BMI (*p <* 0.01) than individuals with normal body weight.

### 3.2. Spatial–Temporal Gait Parameters During the Approaching and Crossing Phases

**Figure 2:**
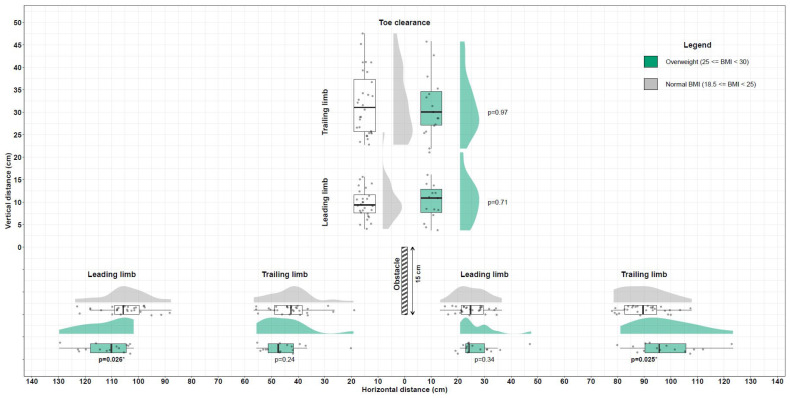
Raincloud plot of the distributions of obstacle clearance parameters measured. *p*: *p*-value of the Wilcoxon rank sum tests; * *p ≤* 0.05.

There were no significant differences between the two groups in any of the spatial–temporal step parameters studied in both the approaching and crossing phases (*p* > 0.05). Figure 2 presents the distributions of horizontal and vertical foot clearance parameters measured in normal body weight (in grey) and overweight young adults (in green) as a raincloud plot. Additionally, individual box plots illustrating the variance of all clearance parameters for both groups are provided as supplementary material.

### 3.3. Leading and Trailing Foot Placement Relative to the Obstacle

The leading foot horizontal distance prior to the obstacle and trailing foot horizontal distance after the obstacle were significantly longer in overweight individuals than in individuals with a normal body weight (*p* = 0.03 and *p* = 0.02, respectively).

There was no significant difference between the two groups in the trailing foot horizontal distance prior to the obstacle and leading foot horizontal distance after the obstacle (*p* > 0.05) (Table 1 and Figure 2).

### 3.4. Leading and Trailing Vertical Toe Clearance During Obstacle Crossing

There were no significant differences between the two groups in the leading and trailing vertical toe clearance during obstacle crossing (*p* > 0.05).

### 3.5. Obstacle Contacts

There was no contact with the obstacle during all trials in both groups.

## 4. Discussion

The aim of this study was to compare spatial–temporal gait parameters, during an obstacle crossing task in normal-body-weight and overweight young adults. Therefore, this study makes a distinct contribution by specifically focusing on overweight individuals, a group often combined with or overshadowed by obesity research in the existing literature. Our hypothesis that overweight impacts gait behavior during obstacle crossing was partially confirmed as only leading foot horizontal distance prior to the obstacle and trailing foot horizontal distance after the obstacle were impacted, and no trips occurred. No changes were observed in the spatial–temporal parameters during both the approaching and crossing phase.

Compared to the individuals with normal body weight, overweight individuals significantly increased the leading foot placement distance prior to the obstacle and the trailing foot placement distance after the obstacle crossing. Considering that vertical clearance parameters are critical outcomes to assess the risk of tripping over an obstacle (e.g., [18,19,37,38,39]), our results suggest that overweight young male adults do not seem to have an increased risk of trips during the obstacle task compared to their normal-weight peers in those specific experimental conditions (crossing an expected 15 cm high obstacle at a self-selected speed).

This foot placement behavior observed in overweight young adults could represent an adaptive, effective and safe obstacle crossing behavior. Considering the vertical foot clearance as the safety margin in the obstacle crossing task, an unchanged clearance does not lead to a higher risk of tripping [39]. Moreover, no participants touched the obstacle during the experimental trials, suggesting that the behavior of overweight individuals is safe when avoiding obstacles. Potentially increasing horizontal distances is a behavior in overweight individuals to maintain dynamic balance (as no difference was observed in double-support time). It could be of interest for future studies to assess the foot trajectory while crossing the obstacle to identify potential differences between overweight and normal-body-weight participants regarding their strategies to maintain toe clearance [40] at different obstacle heights.

Our results revealed no significant group differences in the spatial–temporal gait parameters measured during the approaching and the crossing phase. In other words, both normal-body-weight and overweight young adults had a similar step velocity, length, width and duration and double-support time during the two steps before the obstacle and crossing step. Previous studies reported that step crossing velocity was reduced in obese individuals compared to normal-body-weight individuals. Moreover, the higher the BMI, the lower their velocity during obstacle crossing (reduction of 15.5%, 26.9% and 31.7% for overweight/Class I obesity, Class II obesity and Class III obesity, respectively [15]). In addition, the studies showed reductions in leading leg step length (14%), cadence (13.6%) and single-limb support (relative to a single gait cycle, 6.1%) in obese individuals compared to normal-body-weight individuals, while leading step width (37.4%) and double-limb support increased (relative to a single gait cycle, 24.5%) [13]. When looking at gait phases, Gill. 2019 [15] showed that the step crossing swing phase was reduced in all overweight/obesity classes compared to normal-body-weight individuals (9.7%, 11.4% and 12.7% for overweight/Class I obesity, Class II obesity and Class II obesity, respectively).

One may argue that the gait adaptations observed in obese individuals are caused by higher body mass [2]; hence, individuals with obesity need to increase their base of support for a greater amount of time relative to a single gait cycle [13] and reduce step velocity and length to successfully complete the gait task. A shorter single-limb support during the crossing phase might enhance the risk of tripping over the obstacle, to cross it “more quickly and with less assurance” [13] even if the step crossing speed was reduced. However, in the specific context of crossing a 15 cm high obstacle in laboratory settings, the results of our study showed that overweight individuals do not adopt a similar spatial–temporal gait behavior compared to those with Class I, Class II and Class III obesity. The functional and balance impairments associated with being overweight are probably less deleterious than those associated with obesity [41,42].

Our study investigated overweight individuals, with lower body mass and BMI compared to obese individuals, and overweight individuals are able to adapt their gait behavior when crossing a 15 cm high obstacle at a self-selected pace, which simulates crossing a typical curb height in Brazil [43]. It is possible that the experimental obstacle crossing task was insufficiently demanding to reveal BMI-related changes in foot clearance parameters in overweight individuals due to their lower functional and balance impairments, as well as the likelihood that they perform this task very routinely. As previously mentioned, the obstacle height represents a typical curb height in Brazil [43]. Further studies should consider various task-related factors that have previously been shown to significantly impact foot clearance parameters. These obstacle crossing conditions that also resemble real-life situations could include crossing one or multiple obstacles [44,45] of different heights [39,44,46,47,48] at different walking speeds [49], under various lighting conditions [50,51], while wearing different footwear [46,52] or orthotic bracing [53], during single-task (obstacle crossing only) and dual-task conditions (obstacle crossing with concurrent cognitive or motor task) [54,55,56,57,58].

To the best of our knowledge, this is the first work that compared obstacle crossing during walking in normal-body-weight and overweight young adults. Also, this study is the first to assess BMI influence through obstacle clearance parameters, critical parameters of fall risk during obstacle crossing [16,17]. However, these changes do not seem to increase overweight individuals’ risk of trips and falls when crossing an obstacle. The present findings per se may not reflect what was experienced by individuals with more excessive body weight, such as obese individuals (BMI > 30.0 kg/m^2^). It is furthermore probable that excess body weight would have a greater impact in older adults, as previously reported in other gait tasks [59,60].

The present findings indicate that overweight young male adults demonstrate adaptive gait adjustments during obstacle crossing without increasing their risk of tripping, suggesting stability is maintained under the specific conditions of this study (crossing a 15 cm high obstacle at a self-selected speed in a controlled laboratory setting). Indeed, even if overweight individuals placed their leading limb further prior to crossing the obstacle and their trailing limb further after crossing the obstacle, toe clearances remained unchanged compared to normal-body-weight individuals. The changes in horizontal clearance could be due to overweight individuals’ willingness to ensure more space to cross the obstacle without reducing their vertical clearance. Additionally, the changes in foot placement without a change in vertical clearance imply a different foot motion over the obstacle. Overweight individuals might have a lower foot elevation during the beginning of the swing phase of the leading foot during the crossing step. Nevertheless, this interpretation remains speculative, and it would be of interest for future studies to investigate not only foot horizontal and vertical clearances relative to the obstacle but also the entire foot trajectory during the crossing steps, as was carried out in a recent study [39]. Finally, a relevant aspect that has not been addressed in this or previous studies is the investigation of muscle parameters. Obstacle crossing requires greater neuromuscular activation (e.g., increased knee flexor activation) compared to walking on flat ground [61], and obese individuals have reduced strength normalized by body mass compared to normal-weight individuals [62]. As a result, it seems essential for future studies to couple spatial–temporal and clearance data during obstacle crossing with muscular data in order to better interpret potential limitations in this population when performing this task. However, the limits stated above should caution readers not to draw conclusions too easily or to misinterpret the results.

To better understand whether and how excess body weight impacts obstacle crossing during walking, future studies are necessary to investigate different BMI, age and sex (as women exhibit different gait behavior on flat ground [63] and inclined walking [64]) groups of participants. Studying the influence of BMI on obstacle crossing behavior across various demographic groups would provide a more comprehensive understanding of how BMI affects the functional capacities of these distinct demographic groups. Understanding the distinct gait adaptations in overweight individuals is essential for designing targeted interventions that support their balance and mobility as they transition across BMI categories, whether moving toward lower BMI ranges during weight loss programs or increasing within different obesity classes. These insights could guide the development of training programs aimed at improving balance and reducing fall risk in overweight populations. Such programs may include exercises focusing on dynamic balance, gait stability and obstacle negotiation under various real-world conditions, such as uneven surfaces or varying obstacle heights.

## Figures and Tables

**Table 1:** Group differences regarding socio-demographic and anthropometric characteristics, spatial–temporal gait parameters and obstacle clearance parameters.

Condition	Parameter	18.5 ≤ BMI < 25 kg/m^2^			25 ≤ BMI < 30 kg/m^2^			*W*	*p*	*Effect Size* *(VDA)*
		*n*	Mean ± SD	Median [IQR]	*n*	Mean ± SD	Median [IQR]			
Socio-demographic	Age (years)	28	28.3 ± 6.12	27.9 [8.25]	15	30.6 ± 5.39	31 [6]	154	0.156	0.367 [0.218; 0.546]
Anthropometric	Body height (m)	28	1.76 ± 0.0666	1.77 [0.113]	15	1.78 ± 0.0618	1.77 [0.09]	187	0.566	0.445 [0.278; 0.625]
Anthropometric	**Body mass (kg)**	**28**	**70.8 ± 6.78**	**70.8 [9.41]**	**15**	**86.5 ± 6.15**	**87.9 [5.3]**	**13**	**5.51 × 10^7^**	**0.031 [0.0151; 0.0623]**
Anthropometric	**BMI (kg/m^2^)**	**28**	**22.8 ± 1.72**	**23.5 [1.55]**	**15**	**27.4 ± 1.41**	**26.9 [2.4]**	**0**	**9.22 × 10^8^**	**0 [0; 0]**
Spatial–temporal gait	Stride length (cm)	28	129 ± 9.85	127 [15.2]	15	135 ± 10.8	139 [16.9]	141	0.0808	0.336 [0.195; 0.513]
parameters	Stride width (cm)	28	10.5 ± 2.49	10.2 [2.41]	15	11.8 ± 2.28	11.6 [3.46]	147	0.112	0.35 [0.206; 0.528]
Approaching phase	Stride double-support time (s)	28	0.361 ± 0.0512	0.35 [0.0567]	15	0.344 ± 0.0447	0.333 [0.0533]	263.5	0.177	0.627 [0.447; 0.778]
	Stride duration (s)	28	1.1 ± 0.116	1.09 [0.0742]	15	1.09 ± 0.0986	1.05 [0.12]	240	0.452	0.571 [0.391; 0.735]
	Stride velocity (cm/s)	28	119 ± 14.6	119 [14.1]	15	125 ± 15.8	123 [18.3]	160	0.21	0.381 [0.228; 0.561]
Spatial–temporal gait	Step length (cm)	28	72.1 ± 9.18	71.7 [12.7]	15	75.3 ± 8.59	78.4 [13.4]	160	0.21	0.381 [0.228; 0.561]
parameters	Step width (cm)	28	10.1 ± 2.7	9.45 [3.32]	15	10.6 ± 3.4	11.1 [3.37]	175	0.384	0.417 [0.256; 0.598]
Crossing phase	Step double-support time (s)	28	0.211 ± 0.033	0.207 [0.0317]	15	0.204 ± 0.0359	0.2 [0.0483]	235.5	0.524	0.561 [0.38; 0.726]
	Step duration (s)	28	0.636 ± 0.0547	0.645 [0.0683]	15	0.623 ± 0.0813	0.59 [0.0933]	249.5	0.32	0.594 [0.413; 0.753]
	Step velocity (cm/s)	28	115 ± 17	113 [22.6]	15	123 ± 21.3	125 [26.5]	158	0.192	0.376 [0.225; 0.556]
Obstacle clearance	Trailing foot—horizontal distance prior to obstacle (cm)	28	42.4 ± 8.77	42.7 [10.4]	15	45 ± 9.19	47.5 [9.54]	163	0.239	0.388 [0.234; 0.569]
parameters	**Trailing foot—horizontal distance after obstacle (cm)**	**28**	**90 ± 8.21**	**89.6 [11.9]**	**15**	**98.1 ± 11.6**	**95.7 [15.5]**	**122**	**0.0245**	**0.29 [0.164; 0.46]**
	Trailing foot—toe clearance (cm)	28	32 ± 7.07	31.1 [11.6]	15	31.7 ± 6.61	30 [7.51]	208	0.97	0.495 [0.32; 0.671]
	**Leading foot—horizontal distance prior to obstacle (cm)**	**28**	**105 ± 8.91**	**106 [9.28]**	**15**	**112 ± 8.88**	**110 [13.4]**	**123**	**0.0263**	**0.293 [0.166; 0.463]**
	Leading foot—horizontal distance after obstacle (cm)	28	25.1 ± 5.75	24.8 [8.3]	15	27.3 ± 6.95	24.2 [7.01]	172	0.343	0.41 [0.25; 0.591]
	Leading foot—toe clearance (cm)	28	10.1 ± 4.27	9.38 [4.04]	15	10.4 ± 4.67	10.9 [5.18]	195	0.715	0.464 [0.294; 0.643]

Note: IQR—interquartile range; SD—arithmetic standard deviation; VDA—Vargha and Delaney’s A statistic. Blue background—negligible effect size; green background—small effect size; yellow background—moderate/medium effect size; red background—large effect size.

## Data Availability

The datasets used and/or analyzed during the current study are available from the corresponding author on reasonable request.

## Supplementary Materials

The following supporting information can be downloaded at: https://www.mdpi.com/article/10.3390/s24237867/s1, Figure S1: Box-plots of Clearance Parameters Across Normal Body Weight and Overweight Groups.
