# Supplementary figures and images for: Impact of Overweight on Spatial–Temporal Gait Parameters During Obstacle Crossing in Young Adults: A Cross-Sectional Study
Source: Sensors (Basel). 2024 Dec 9;24(23):7867. doi: 10.3390/s24237867 (PMC11644976; doi:10.3390/s24237867)

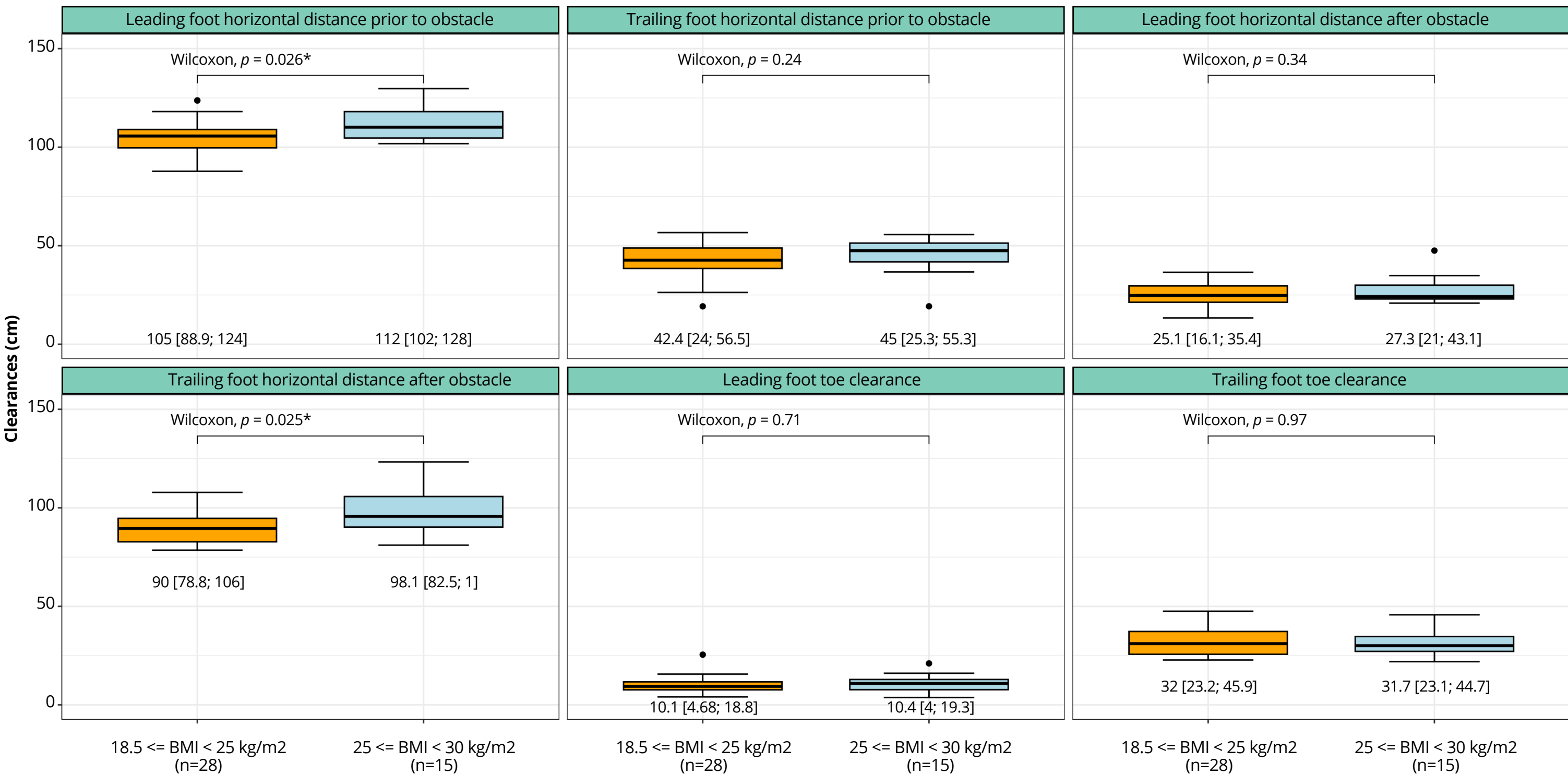

Supplement: Supplementary file 1 [file sensors-24-07867-s001.zip › sensors-3301782-supplementary.pdf]
